# Motivating youth to learn STEM through a gender inclusive digital forensic science program

**DOI:** 10.1186/s40561-022-00213-x

**Published:** 2023-01-06

**Authors:** Eoghan Casey, Jennifer Jocz, Karen A. Peterson, Daryl Pfeif, Cassy Soden

**Affiliations:** 1 Cyber Sleuth Science Lab, New Orleans, USA; 2 Education Development Center, Waltham, USA; 3 National Girls Collaborative Project, Seattle, USA

**Keywords:** Smart learning environment, STEM education, Gender inclusive instruction, Problem based learning, Cybersecurity, Digital forensic science, Investigate and Decide Learning Environment

## Abstract

This paper describes the design, implementation and research of the Cyber Sleuth Science Lab (CSSL), an innovative educational program and supporting virtual learning environment, that combines pedagogical theory, gender inclusive instruction strategies, scientific principles/practices, gamification methods, computational thinking, and real-world problem solving. This program provides underrepresented youth, especially girls, with digital forensic knowledge, skills and career pathways, challenging them to explore complex social issues related to technology and to become cyber sleuths using real-world digital forensic methods and tools to solve investigative scenarios. Students also learn about related careers while improving their cyber street smarts. The CSSL incorporates additional “outside of the computer” activities to strengthen students’ engagement such as structured in-classroom discussions, mock trials, and in-person interactions with practitioner role models. The CSSL was piloted in various forms to assess the suitability for in-school and out-of-school settings, and the students predominantly represented racial minorities. Research in this project relied on a mixed methods approach for data collection and analysis, including qualitative and quantitative methods, reinforced using learning analytics generated from the students clicking through the interface and interacting with the system. Analysis of gathered data indicate that the virtual learning environment developed in this project is highly effective for teaching digital forensic knowledge, skills, and abilities that are directly applicable in the workplace. Furthermore, the strategies for gender inclusive STEM instruction implemented in CSSL are effective for engaging girls without being harmful to boys’ engagement. Learning STEM through digital forensic science taps into girls’ motivations to address real-world problems that have direct relevance to their lives, and to protect and serve their community. After participating in the educational program, girls expressed a significantly greater increase in interest, relative to boys, in learning more about careers related to digital forensics and cybersecurity.

## Introduction

Cybersecurity and cybercrime have become part of everyday life, creating diverse challenges and opportunities. Schools are experiencing a wide variety of problems involving technology, including cyberbullying and data breaches. Local governments, public institutions and schools are being disrupted by ransomware. Critical infrastructure are being targeted by sophisticated cyberattacks, creating significant actual or potential harm to the environment, economy, and human life (Casey & Nikkel, 2020). People are being targeted in their homes by online fraudsters and hackers. Investigating and solving these complex problems requires scientific reasoning, technical knowledge, computational thinking and mathematics all based on digital evidence, combined with social considerations (Casey, 2011). These problems are growing rapidly and there is a major shortage of qualified job candidates in Cybersecurity, which includes Digital Forensic Science, with 2.72 million unfilled positions in 2021 ((ISC)^2^, 2021). Responding to this growing need, some states are creating standards for high school cybersecurity programs, which include digital forensics and incident response (Nevada Department of Education, 2018; K12 Computer Science Framework Steering Committee, 2016; Perhach, 2018).

Notably, relatively few women enter this dynamic workforce sector (Center for Safety and Education, 2019; (ISC)^2^, 2021), representing just 25% of the workforce in 2021, which is in stark contrast to other forensic science disciplines which attract many more women than men (Barbaro, 2019). When women are not represented in the workforce, the field risks losing the diverse ideas and inputs that they bring with them (Ashcraft et al., 2012; Hill et al., 2010). This problem presents an opportunity for educators to meet the need for more traditionally underrepresented professionals in STEM related fields (Gonzalez, 2012) by providing the populations with the knowledge and skills needed to pursue careers in Digital Forensic Science and Cybersecurity, and to address the increasing number and severity of cyberattacks and cybercrimes.

Prior research specific to cybersecurity education for high school students in 1-week GenCyber summer camps found game based learning to be very effective (Jin et al., 2018). The GenCyber summer camp did not focus on implementing gender inclusive instruction, and boys expressed higher enjoyment and interest than girls. Research into factors contributing to girls’ engagement in informal STEM education, although not related to cybersecurity, found nine design attributes that are most effective for engaging girls without being harmful to boys’ engagement (Dancstep & Sindorf, 2018). These design attributes include the look-and-feel (engage), diagrams showing how to use an interactive element (guide), group participation and peer learning (collaboration and communication).

A primary research questions in this work is do underrepresented youth, especially girls, report being more interested in STEM after learning through realistic digital forensic investigations? A core component of this work was to evaluate the effectiveness of the CSSL for teaching digital forensic knowledge, skills, and abilities that are directly applicable in the workplace, and for motivating underrepresented youth, particularly girls, to pursue careers in Digital Forensic Science and Cybersecurity in particular, and computer science in general. The mystery solving focus of the CSSL provides ample opportunity to evaluate performance including comprehension, level of engagement, and knowledge development.

To address the growing need for underrepresented populations, especially girls, in digital forensic science and cybersecurity, this project combined state of the art learning science, gender inclusive instruction, innovative technology, and engaging subject matter to advance STEM education in informal settings. The novel contributions and significant outcomes of research and development performed in this project include:

*Pedagogical model*: Designed and developed a flexible, powerful, adaptable model and working proof-of-work learning environment that can be applied broadly to teaching other STEM disciplines.

*Implementation strategies for gender inclusive STEM instruction*: Constructed a set of strategies for researchers/developers to follow when implementing gender inclusive instruction in informal STEM education.

This paper provides motivation for teaching STEM through digital forensic science, describes the design and implementation of the CSSL, and presents the research method, data gathering and outcomes.

## Motivation

Digital Forensic Science was selected as the focus for this program in part because it is deeply rooted in STEM, combining scientific practices with in-depth analysis of technology, including hypothetico-deductive reasoning and computational thinking. In addition, Digital Forensic Science is inherently inquiry-based which is central to effective STEM education (Kim, 2016), and is strongly compatible with existing standards for science and technology education. In addition, the multidisciplinary nature of these domains, encompassing social issues such as justice, safety, privacy and ethics creates opportunities for students to think about and engage with science and technology from multiple perspectives. Prior work indicates that computer science education which integrates social justice and shows underrepresented youth how they can address problems in their community increases their interest in computer science education (Denner et al., 2015).

In particular, Digital Forensic Science and Cybersecurity are oriented to helping others deal with real-world problems (Boucher et al., 2017) and tap into the potential that young women “may be more attracted to science and technology related careers that involve complex social issues and societal impact” (Modi et al., 2012). Since one of the strongest predictors of young women’s interest in computing education is the extent to which they see value and relevance in computing, teaching STEM in the context of real-world problems that have direct relevance to students’ lives is vital for increasing levels of interest (Ashcraft et al., 2012; Christensen & Knezek, 2015). In addition, Digital Forensic Science and Cybersecurity provide youth with opportunities to protect and serve their community using science and technology, which provides strong motivation for learning STEM (Diekman et al., 2015).

Dealing with problems that youth find personally relevant, have social impact, and help others, the CSSL is designed to engage underrepresented youth, especially girls, in STEM learning and career pathways, while exploring complex issues associated with cybercrime (a reason to care). In particular, the CSSL supports girls with a responsive instructional environment, collaborative problem solving, and role models who are experts in the domain (Denner et al., 2015; Jethwani et al., 2016; Koch et al., 2015; Trujillo et al., 2015). Through immersive goal-based learning experiences, combining online and in-person classroom elements that cast students in the role of cyber-investigators, the CSSL helps students develop scientific reasoning, problem solving, technical knowledge, computational thinking, engineering, mathematics foundations, and practical skills, and equips them to pursue careers related to science, technology, engineering, mathematics and computing (STEM).

## Design

Creating the foundation necessary to support effective personally meaningful STEM education for underrepresented youth, particularly girls, was a significant effort in this project, combining pedagogical theory, gender inclusive instruction strategies, scientific practices, gamification methods, computational thinking, and real-world problem solving.

### Model for inquiry-based pedagogical design

The CSSL is a blended learning program that combines a sophisticated virtual learning environment hosted in the cloud with rich goal-based scenarios and supporting educational resources to fuel in-classroom instructional activities and career pathway exploration. Specifically, the CSSL was developed using a problem based learning (PBL) pedagogical strategy, constructed around engaging goal-based scenarios, extending prior learning science work. Specifically, the design and development of the CSSL involved critical evaluation of an instructional model called Investigate and Decide Learning Environment (IDLE). The inquiry-based structure was adapted to teach Digital Forensic Science and Cybersecurity, and was augmented with the strengths of cognitive apprenticeship and case-based reasoning. In addition, this work reinforces and refines the design and implementation of inclusive STEM instruction. At each step in the structure of the investigative scenarios the project team considered how the principles of gender inclusive instruction could be incorporated, as elaborated on in the next section.

The IDLE model is suitable for scientific domains that involve evidence based conclusions (Bell, 1996, 1998). Combining this with cognitive apprenticeship and case-based reasoning integrates collaborative learning, structuring complementary cases, and practicing skills in diverse contexts (Collins et al., 1989).

Research shows that we learn best through a combination of theory and practice in a realistic context (Brown et al., 1989). Theory is inert until it is applied to a realistic situation, and practice is shallow without the influence of theory (Norman, 1993). In addition, students learn science and critical thinking more effectively when applying knowledge and skills to authentic situations (Duran & Dökme, 2016). Students are motivated by goal accomplishment, including intrinsic satisfaction of solving a case, and earning merit for acquiring skills (Schank et al., 1994).

The IDLE model emphasizes situated learning, but neglects the importance of students learning from each other and of designing investigative scenarios that are engaging and effective. Research indicates that practicing skills in a variety of contexts is the key to knowledge transfer.

As students learn to apply skills to more diverse problems and problem situations, their strategies become freed from their contextual bindings (or perhaps more accurately, acquire a richer net of contextual associations) and thus are more readily available for use with unfamiliar or novel problems

(Collins et al., 1989)

Careful attention is required to select and structure multiple instructional scenarios in a way that helps students arrange them in their minds, make generalizations, and understand important aspects (Kolodner, 1994; Schank, 1990, 1994; Schank & Abelson, 1977).

### Gamification: creating narrative structure and casting students as cybersleuths

The CSSL extends and bolsters the IDLE model using gamification with carefully selected instructional scenarios structured to help students apply scientific practices and technical skills in diverse contexts to help students abstract and apply what they learn in varying situations to solve differing problems. The CSSL casts students as cybersleuths with a mission to accomplish, using cyber-pseudonyms to play the role and maintain anonymity. Missions are structured within an immersive virtual learning environment, steadily increasing in difficulty levels, with recognition for achieving each level. Student progress is tracked continuously within the CSSL, and is accessible to students to provide visibility into their own achievements and to compare with others as discussed in the data gathering section of this paper.

**Fig. 1:**
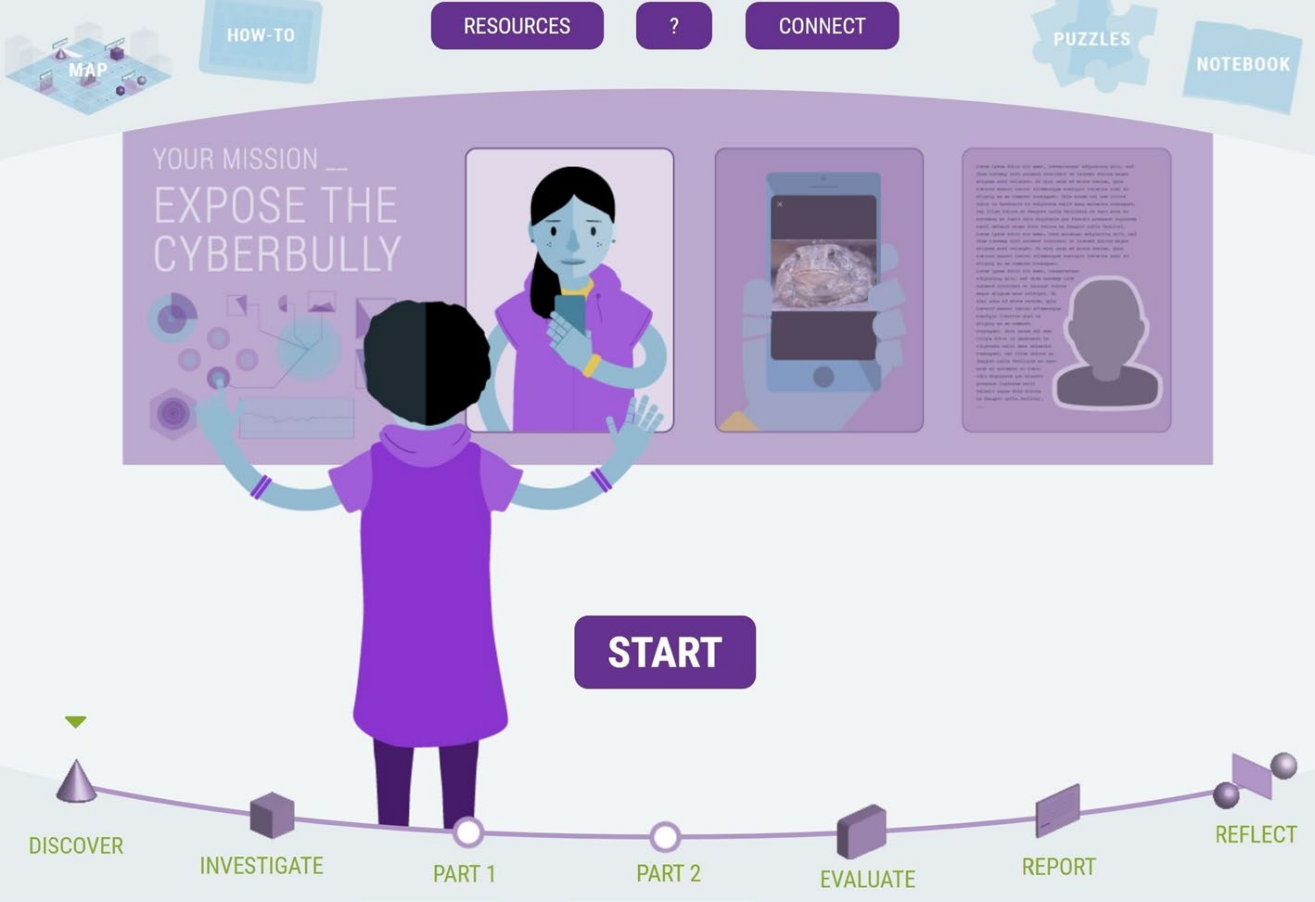
Missions in the CSSL platform provide a cognitive map of an evidence-based scientific investigation process to guide students through each phase

To structure and support problem solving, computational thinking, and hypothetico-deductive reasoning, the formation and execution of an evidence-based scientific investigation is explicit within the CSSL, providing students with a map of the cognitive processes and guiding them through the phases shown in Fig. 1. Each phase has a milestone marker at the bottom of the screen, and a map of the full process is always available in the top left of the screen for ease of navigation. For each mission, the initial Discover phase involves forming an investigative strategy, which is executed during the Investigate phase using digital forensic tools to process and analyze data. For more complex investigative missions, the Investigate phase is divided into parts with separate objectives and assessments. This is followed by the Evaluate phase which requires interpretation of evidence to support a decision. In the Report phase, students are required to compile their observations and conclusions into an objective, logical presentation (written and/or oral). The Reflect phase occurs in the classroom, with a guided group discussion about how students resolved challenges they encountered during the investigation, and about related ethics and social impact such as bias, privacy and justice.

The importance of dramatic structure cannot be underestimated when creating an engaging mission. Aristotle understood the importance of dramatic structure when he wrote “Poetics,” and Laurel elaborated on Poetics when she wrote “Computers as Theatre” (Laurel, 2013). Developing dramatic stories is a time consuming process but is necessary to create effective, engaging and enduring educational content. Missions in the CSSL were constructed with input from youth dealing with issues that are directly relevant to their lives, including cyberbullying, privacy, account hijacking, identity theft, anonymous harassment, unauthorized sharing of personal photographs. Specifically, the following missions were create in the CSSL with associated datasets for students to analyze using specialized software:

- Mission 0 (Deleted Photos): A basic introductory mission involving recovery of deleted files from grandmother’s camera SD card, enabling students to become familiar with all elements of the CSSL.
- Mission 1 (Cyberbullying by Phone): A student is cyberbullied anonymously via her phone, and deletes the upsetting messages. The mission is to recover the deleted messages, determine where the messages were sent from, follow a dead end, and then examine the suspected cyberbully’s phone for evidence of sent messages.
- Mission 2 (Embarrassing Photo): An embarrassing photoshopped photo of a student is sent anonymously to everyone at a school. The student in the photo is very upset and needs help. The mission is to analyze forensic copies of mobile devices to determine who took/sent the photo.
- Mission 3 (Account Hijacking): A student receives a phishing email that tricks her into disclosing her password. Her account is then used to solicit financial donations to a fraudulent charity cause. She needs help proving that she was not responsible for the fraud. The mission is to analyze email headers, server log files, and phishing website to determine where the phishing attack came from (IP address).

Throughout all missions, the CSSL integrates scientific practices and reasoning, technical knowledge and skills, computer science concepts and programming, and social issues and societal impact. Each analytical task within the Investigate phase involves constructing a plan to search the provided data in order to find evidence relevant to the investigation. This plan is then executed applying scientific practices and using real-world digital forensic tools, and the results are extracted to address specific questions the students must answer. Discrete programming tasks teach students to write code for processing data and extracting information.

This immersive experience enables students to rapidly acquire fluency in the skills and knowledge, supported by resources and facilitator supports. Casting students as cybersleuths gives them an active and positive role to play, and creating missions with personally relevant real-world problem-based scenarios gives them a reason to care.

### Framework for implementing gender inclusive instruction

An overarching consideration throughout the CSSL is to bring into practice the principles and strategies of effective gender inclusive instruction in informal STEM education. Specifically, the CSSL codifies eight principles of effective design and instruction, particularly for girls: (1) Engage and Empower, (2) Enculturate, (3) Immerse, (4) Guide, (5) Practice and Iterate, (6) Synthesize, (7) Collaborate and Communicate, and (8) Reflect. These eight principles support and extend the seven SciGirls strategies (Billington et al., 2019), and are further informed by recent research into gender inclusive strategies (Hughes et al., 2020). The successful approach to implementing gender inclusive instruction in STEM education in this project provides a set of strategies for others to follow. Table 1 shows the alignment of these eight principles with specific elements implemented in the CSSL program.

**Table 1:** Implementation strategies for gender inclusive STEM instruction

Design feature	Principle of gender inclusive instruction	Implementation guidance
Gamification	Engage and Empower, Immerse	Create missions that pose personally meaningful problems to help others solve (giving girls a reason to care), using dramatic narrative structure, with defined clues to find and tasks to accomplish in realistic settings, with increasing levels of difficulty
Gamification	Engage and Empower	Cast students in the active positive role of investigator or problem-solver
Gamification	Engage and Empower, Immerse	Create a learning environment that enables girls to form a sense of belonging (inclusive and inviting look-and-feel)
Gamification	Engage, Guide	Make progress visible to teachers and students with live learning analytics, and have students keep track of their own progress using this information (keeping score)
Inquiry-based pedagogical model	Engage and immerse, practice and iterate, synthesize, communicate, and reflect	Embed structure and support for following scientific practices of discovery, inquiry, documentation, evidence evaluation, and communication (present, report, reflect)
Inquiry-based pedagogical model	Immerse, Guide, Enculturate	Integrate STEM core concepts and practices throughout the missions, including considering alternative hypotheses, testing theories, evaluating evidence objectively, documenting examinations and results, and formulating conclusions
Inquiry-based pedagogical model	Guide	Provide robust structured supports for major goals performed together in class
Inquiry-based pedagogical model	Guide, Practice and Iterate	Give students instructions to perform a task (*guide*), then let them try the task in a structured learning environment (*practice*), then demonstrate the task (*guide*), and then let them try the task again (*iterate*)
Inquiry-based pedagogical model	Collaborate and Communicate	Encourage group work and peer-learning for all activities
Inquiry-based pedagogical model	Collaborate and Communicate, Synthesize	Unplugged activities that require students to consolidate and convey what they learned, such as investigative reports and mock trials. It is important to give students the means and opportunity to prepare and refine their communication skills, guiding each other, such as students reviewing each other's reports to incorporate peer learning and writing revision, and recording oral presentations for teachers to provide feedback (*practice*). Ultimately, having students give presentations in class reinforces communication skills and peer-learning (*observing similar others succeed*). Unplugged activities must be built into the program, both for learning and to reduce student fatigue in immersive learning environments
Inquiry-based pedagogical model	Reflect	Create opportunities for students to discuss with teachers and role models how the skills they learned could be applied to real world problems, including current local events where forensic science helped solve societal problems
Inquiry-based pedagogical model	Enculturate, Reflect	Create opportunities for students to interact with role models (students or professionals in the field) to gain real world context, practical insights, career examples, further learning opportunities, and a sense of belonging

It was challenging to find contexts to teach only girls, and many environments in which the CSSL can be beneficial are coed. To deal with this reality, efforts were made to incorporate supports specifically to help girls in coed environments. These supports included increasing in- classroom activities (e.g., mock trial, Socratic circle), group work for peer learning support, relevance of investigative scenarios to their daily lives, young female role-models and in- classroom facilitators (near peers), and educator and facilitator awareness of the importance of providing girls with personal positive feedback and promoting a growth mindset (Denner & Campe, 2018; Trujillo et al., 2015).

**Fig. 2:**
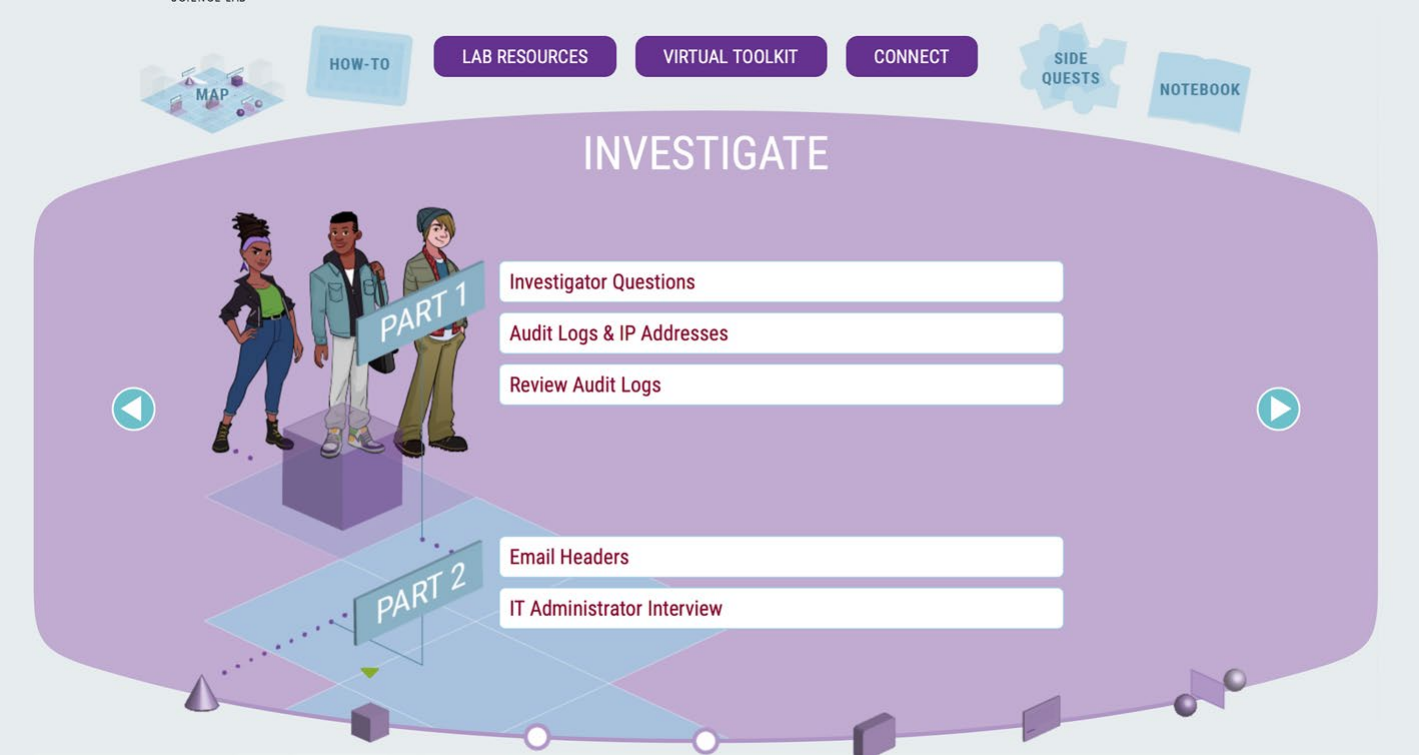
The CSSL platform look-and-feel was updated after 2019 based on student focus group and survey feedback, shown here in Mission 3, which exhibits increased complexity for students to develop and apply what they learn in earlier missions

Research shows that the learning environment must be safe and inclusive, with a look-and-feel that is inviting and allows girls to form a sense of belonging (Hubert, 2014; Sammet & Kekelis, 2016). To create a learning environment with an inclusive and inviting look-and-feel, the CSSL conducted initial student focus groups to guide the design, including character representation, robot/space themes, and color scheme (see Fig. 1 above). In 2019, to evaluate the suitability and success of the resulting design and development, several student groups were surveyed and another focus group was held. Feedback determined that there was a need to better represent diversity and not try to “erase” it by making characters blue. In response to this feedback, the project team developed the redesign of three primary student characters and Dr. Z (the character that assigns missions and provides information and clues to solve the case). The refined look-and-feel of the virtual learning platform is shown in Fig. 2 below.

## Implementation

The project research and development strategy concentrated on informal education settings, iteratively improved based on participant feedback, and stress tested the program to determine whether it could serve the needs of in-school and online only education. Educational settings were selected based on their inclusion of underrepresented youth, predominantly racial minorities in Baltimore, New Orleans, Las Vegas, and a Seattle school district. The CSSL modular design allows for flexibility to adapt materials to different educational settings. This flexibility allowed the program to be tested in a single day session, multiple full-day sessions, and across multiple weeks in different formats. Single day sessions focused on one mission with limited duration in-classroom activities and role model engagement, whereas longer sessions permitted expansion of all aspects of the CSSL program.

- 2017–2018: Focus groups and design sessions, driving intensive development of platform with pedagogical foundation, and curriculum reinforcing scientific principles & practices, and critical & computational thinking.
- 2018–2019: Successful 1-week pilot with 79 students (40% female) conducted simultaneously in four classrooms, as part of a larger summer program serving Baltimore City public school. Lessons learned improved subsequent implementation of principles/strategies of gender inclusive instruction, and realized the educational value of live learning analytics to support teaching visibility into student progress.
- 2019–2020: Stress testing with: (a) an expanded summer program hosted by a Seattle school district, supported by Washington Network for Innovative Careers (WANIC), lasting 3 weeks (78 total hours), with 12 students (50% female), (b) in-school simulation with one hour per day during 5-week summer work-study program, with a total of 32 students (40% female) in two classrooms, and (c) workshops.
- 2020–2021: Super stress testing fully online, incorporated into the curriculum (42 total hours) at two high schools in Las Vegas reaching four teachers and 93 students in grades 9-11 (39% female).

A deliberate process of iterative improvement was followed to incorporate lessons learned from each pilot, including insights from formative evaluations, feedback from participants, and input from advisory board members. This process included student focus groups to improve learning activities and resources, and teacher focus groups to improve teaching materials and tools. In addition to the primary pilots, portions of the CSSL program were presented to Girl Scouts and larger events to obtain feedback and to motivate more girls to consider career and education pathways related to Digital Forensic Science and Cybersecurity.

### Implementation and technology

Throughout the program, students are led by educators who have been familiarized with the CSSL platform and materials. Students work independently and in groups, depending on the task and preference of the educator. Learning supports for students are embedded within the CSSL platform to provide needed technical skills and fundamental scientific and technical concepts. In addition, reference materials are always accessible to student within a resources section of the virtual learning environment. An electronic Notebook is integrated into missions for students to follow scientific practices (e.g., contemporaneous note-taking, documentation), and to capture what they learned for assessments and reporting.

The CSSL is structured to encourage collaborative learning and employs near peer facilitators to enhance student advancement and learning (Denner & Campe, 2018; Denner et al., 2015; Trujillo et al., 2015). When meeting in-person, facilitators support educators and students by circulating in classrooms to address technical issues and student questions.

The technology platform integrated a web-based virtual learning environment with a cloud-based virtual machine laboratory, enabling educators and students to immerse in the CSSL from anywhere. Traditionally, both a web browser and remote desktop application were required to access remote systems and applications, which complicated accessibility. To eliminate barriers to entry, the CSSL made all areas and resources available via a single web interface.

**Fig. 3:**
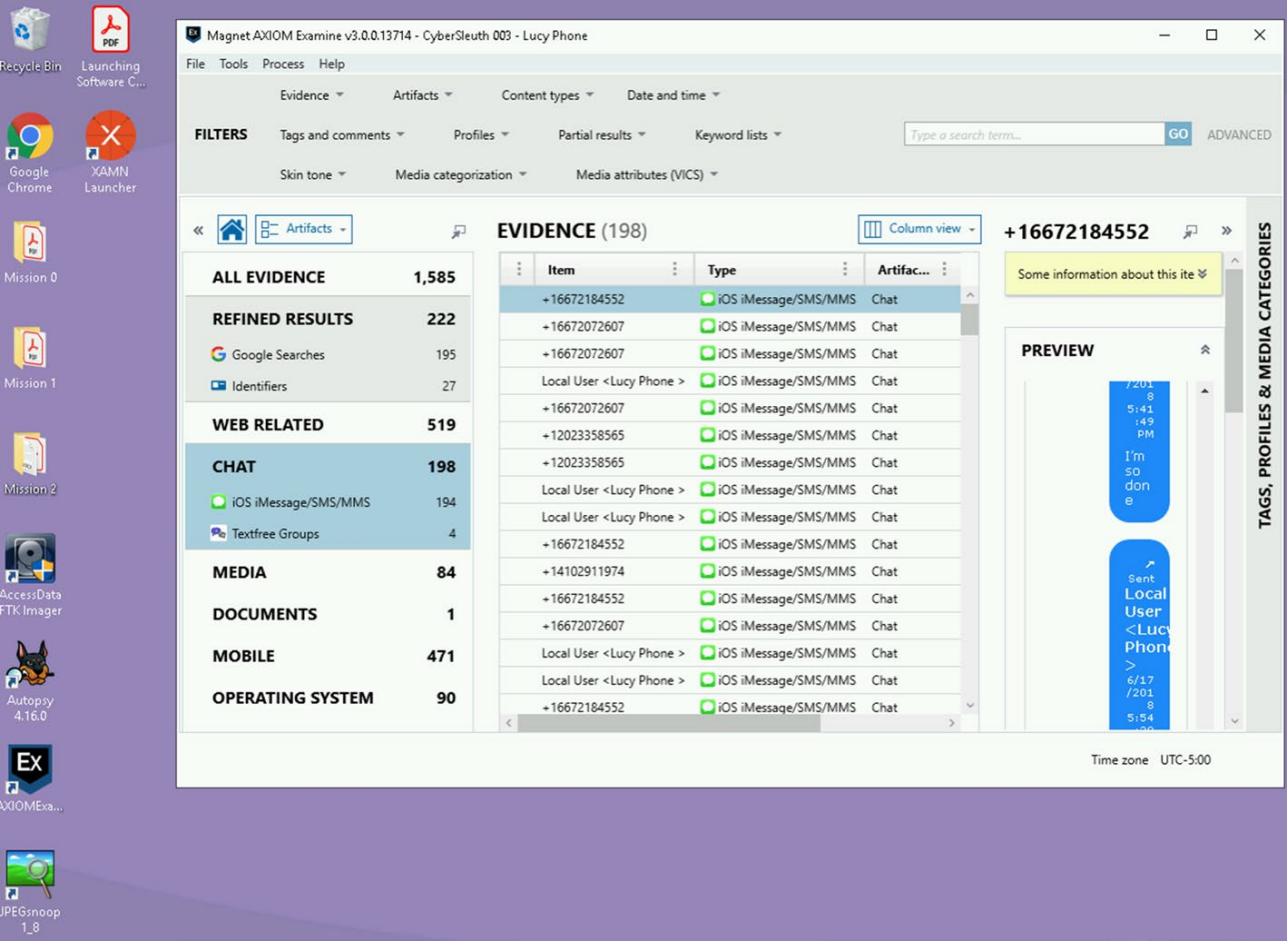
Cloud-based virtual machine laboratory (Virtual Toolkit) with digital forensic tools for students to examine datasets as part of the investigative missions

The virtual machine laboratory provides students with access to professional software tools and realistic investigative challenges to solve as shown in Fig. 3. In the words of the pioneers in situated cognition, people learn best when learning “to use the tools as practitioners use them” (Bell, 1996). Missions involve technology that students use personally, particularly smartphones, increasing their familiarity with the technology and associated risks.

A role model and mentor network of practicing scientists and industry experts, being developed in collaboration with the National Girls Collaborative Project (NGCP) online role model directory, FabFems (https://www.fabfems.org/), gives youth opportunities to interact with adult role models and gain deeper insights into career pathways. Classroom activities provide additional support and role model engagement.

## Research method and data gathering

Research in this project relied on a mixed methods approach for data collection and analysis:

- Qualitative examination of student and teacher interview and focus group data to identify patterns and themes of student engagement, interest, and motivation to learn more, as well as themes of teacher fulfillment and future needs.
- Quantitative analysis of multiple choice and Likert-scaled survey items using descriptive statistics to perform comparisons of student knowledge and skill level before and after completing the CSSL curriculum, and to understand changes in student understanding and interest.
- Qualitative examination of responses to open-ended survey items to identify patterns and themes of strengths and weaknesses in CSSL program.
- Examination of backend metrics to understand patterns of student use and program progression, i.e., learning analytics resulting from the students clicking through the interface, interacting with the system, and completing tasks.

### Data gathering

Multiple methods were used to gather data from students, and each instructional module contained formative assessments to determine whether students performed technical and problem solving tasks correctly, and summative assessments to determine whether they understood the overall mission. When time permitted, students wrote reports and presented their work in class. These written reports and oral presentations provided valuable insights into student understanding, and gave students an opportunity to develop their communication skills when presenting evidence-based conclusions. Students were surveyed before and after the program to assess their attitudes, knowledge and experiences.

In 2019, the CSSL platform was augmented to gather detailed backend metrics to study how students use the platform, where they encounter challenges, and what areas require additional scaffolding. Preliminary analysis of these data was performed in this work, and more in-depth analysis is planned in future work to refine the effectiveness of the CSSL platform. Data sources included field notes from classroom observations, notes from focus groups and interviews, multiple-choice and Likert-scale surveys, student artifacts, and backend user metrics.

For all in-person pilots, one or more of the project team members were on-site to observe classroom activities, and field notes were compiled. The final online pilot required remote observations due to COVID constraints, which was constrained by policy and technology barriers, with most visibility into one of the two classrooms. These observations were looking for students’ level of engagement, peer-learning and group work dynamics, interactions between students and educators (teachers and facilitators), and technical difficulties using the virtual learning environment.

On the last day of the program, students in all locations completed a survey focused on their overall experience in the program and its impact on their knowledge and awareness of digital forensic science and cybersecurity, as well as their awareness of and interest in related careers. Multiple-choice and Likert-scale survey questions addressing student interest, knowledge, and motivation to learn more, were developed by the project team and student surveys were administered pre and post pilots.

Student focus groups were conducted towards the end of the program to gain a deeper understanding of students’ experience in the program. Students also filled out a short reflection sheet during cycles 1 and 2 when they completed the missions to gather their thoughts about the specific digital module they worked on. Teacher focus groups were held at the end of the program to review challenges and successes, capture lessons learned, and identify gaps to be filled. All participating teachers and facilitators were interviewed either during site visits or by phone either near or shortly after the end of the program to gather information about their experience implementing CSSL. Instructors and facilitators also completed a survey during the first implementation of the program.

Note that facilitators were not interviewed or surveyed during the final cycle of implementation because the COVID-19 pandemic prohibited bringing in outside facilitators. Student artifacts, including final investigative reports in earlier pilots, increased for the Seattle 2019 pilot with class timeline creation, mock trial preparation, and written artifacts. The Seattle 2019 pilot also Individualized Learning Plans (IEP) and exit tickets to track student achievements.

### Backend metrics

Backend metrics from the project virtual platform/curriculum that includes embedded assessment data of student knowledge and skills, were recorded to study how each student was progressing through the CSSL curriculum. The research team used the backend metrics as a data source to look at student performance in the following ways:

- Student performance on discrete tasks within each mission, which require the student to examine digital evidence. Backend metrics capture when students accessed the virtual toolkit to examine data, when they completed each task, and how successfully they performed the task.
- For each discrete task, the student is required to provide information about the digital evidence in order to solve the case. Backend metrics capture student answers and record whether the answer is correct or incorrect.
- Whether or not the student accessed additional instructional resources.

This quantitative data is useful for addressing research questions, and provides the type of transparency that is a necessary requirement for progress tracking and classroom management in computer-mediated instruction contexts, especially distance learning.

### Teacher use of learning analytics

**Fig. 4:**
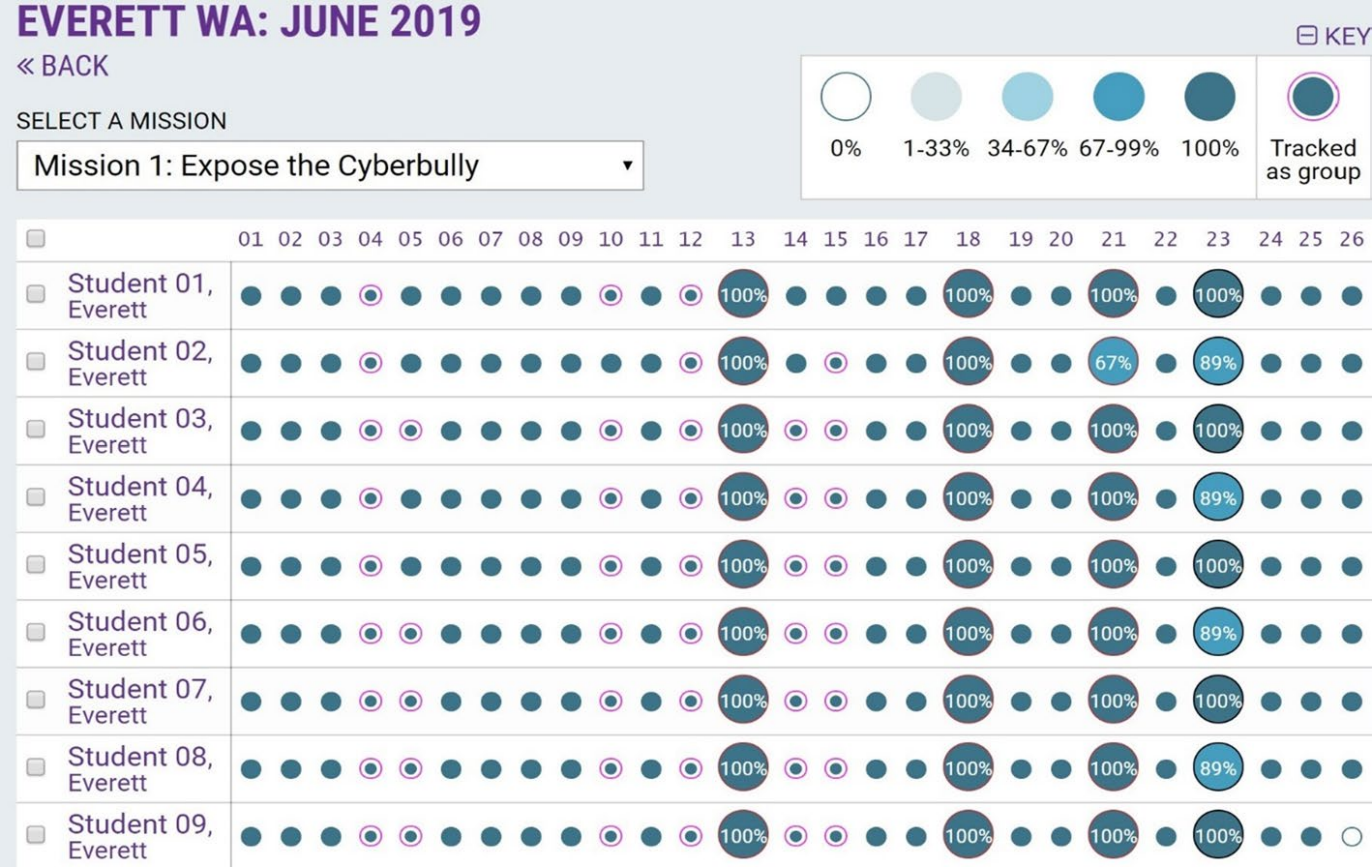
Teacher Dashboard with learning analytics for all students in a session, used to manage learning activities and track progress of individuals and groups

The initial intent was to use backend metrics to produce learning analytics just for research. However, immediately following the Baltimore 2018 pilot, while reviewing the backend metrics during a teacher focus group, it became clear that teachers would have benefited from having visibility into the learning analytics in real-time while they were helping students complete activities. The teachers specifically requested that the learning analytics be made accessible in a way that they could use while teaching. In response, the project team developed the Teacher Dashboard (Fig. 4) which displays learning analytics to provide visibility into student participation and progress in each mission, performance on formative assessments and summative assessments, as well as to enable tracking of students working in pairs or groups. This resource enables teachers to keep their finger on the pulse of student learning and to steer the class through challenges together.

**Fig. 5:**
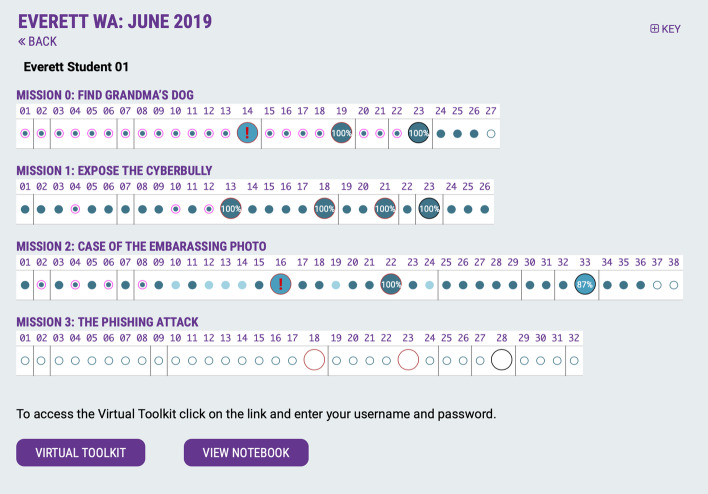
Student Personal Dashboard with learning analytics for all missions, allowing each student to see their own progress and compare with others

In Fig. 4, each row in the Teach Dashboard shows the progress of a student through a given mission, with small solid circles indicating accomplishments that students on their own, and red outlined small circles indicating accomplishments that students accomplished together as a group or through teacher-led activities. The student assessments embedded within the mission are represented by larger circles with percentage success. Shading within each circle shows the level of completion as indicated in Key (top right). Exclamation points within a circle (see Fig. 5 below) are used to alert that a student needs help, prompting the facilitator or teacher to provide additional assistance.

### Student use of learning analytics

Based on the usefulness of the Teacher Dashboard to provide transparency and insight into student progress, this tracking mechanism was used to provide students with their Personal Dashboard, enabling each student to see their own progress (Fig. 5). Teachers used this tracking mechanism throughout the CSSL program to have students view their own individual progress and see what they still need to improve or complete. Students could use this tracking mechanism to compare their progress with others. In Fig. 5 showing data from 2019, the Mission 3 was not yet integrated into the platform to populate learning analytics, and was completed in-class. Mission 3 is now integrated into the platform to capture learning analytics.

## Analysis and outcomes

Student and teacher interview notes and open-ended survey responses were analyzed using a constructivist grounded theory approach (Charmaz, 2006) to identify patterns and themes related to themes of student engagement, interest, and motivation to learn more, as well as themes of teacher fulfillment and future needs. Quantitative analysis of normalized pre-post survey responses was performed, comparing girls with boys, to produce descriptive statistics to understand changes in student understanding and interest.

Backend metrics were aggregated and analyzed to study student progress and performance through tasks and assessments, enabling analytics-reinforced research. A synopsis of analysis of all data collected during the project is provided in Table 2. Some data was limited during the initial development of the project as the platform and program were still under development. The 2020–2021 pilot in Nevada was complicated by COVID constraints, and efforts are ongoing to fill gaps in visibility of student engagement and performance.

**Table 2:** Summary of results (*Technology posed an issue in collecting releases from students and their family which impacted our ability to also collect student artifacts from the students.)

Venue	Results	Results				
	Sample	Observations	Surveys	Interviews	Backend Metrics	Student Artifacts
Baltimore 2017 Design & Development	12	Enthusiastic, focused, high engagement, constructive feedback	High increased interest, knowledge, and motivation to learn more	Benefited from learning scaffolding (written & videos), learned more about complexities in computers + society	Under development	Scenario selections, design look & feel
NOLA 2017 Design & Development	30 (4 + 26)	Enthusiastic, focused, high engagement, constructive feedback	High increased interest, knowledge, and motivation to learn more	Group discussions	Testing	Constructive feedback on platform and program
Baltimore 2018 Implementation	79	Enthusiastic, focused, high engagement, constructive criticisms	High increased interest, knowledge, and motivation to learn more	Engaging instruction, Felt like a professional worker, can use what they learned to solve problems and help others, benefited from learning scaffolding (written & videos)	Limited data	Final reports
NOLA 2019 Implementation	17	Enthusiastic, focused, high engagement, constructive criticisms, active teamwork, engagement w/ near-peers	Moderate-high increased interest, knowledge, and motivation to learn more	Benefited from learning scaffolding (written & videos), liked interaction w industry role models	Variable task completion, low percentage performance, low average final scores	N/A
Seattle 2019 Stress Testing	12	Enthusiastic, focused, high engagement, constructive criticisms	High increased interest, knowledge, and motivation to learn more	Benefited from learning scaffolding (written & videos), wanted missions to be less childish	100% task completion, high percentage performance, high average final scores	Student reflections, student reports, class timeline
Baltimore 2019 Stress Testing	32	Tired, distracted, low engagement, low feedback	Moderate increased interest, knowledge, and motivation to learn more	Limited data	High percentage task completion, variable percentage performance, medium average final scores	Limited data
Las Vegas 2020 Super Stress Testing	98	Remote, distracted, sporadic engagement, limited feedback	High increased interest, knowledge, and motivation to learn more	N/A	Variable task completion, low percentage performance, low average final scores	Unavailable*

### Comparative analysis of pre-post surveys

The paired-samples t-test comparing by gender of pre-post survey results related to research questions are presented in Table 3 below.

**Table 3:** Paired-samples t-test comparison by gender of students’ understanding of and interest in digital forensic science and related careers based on retrospective pre-post survey questions

Gender	Statement	Mean difference (Scale: 1–4)	SD	p-value	Effect size (d)	Effect size strength
Female (n = 63)	I am interested in computer science topics	+ 0.317	0.839	0.004	0.38	Small
	I am interested in digital forensics topics	+ 0.508	0.840	< 0.001	0.60	Moderate
	I am interested in using digital forensic applications to solve problems	+ 0.571	0.875	< 0.001	0.65	Moderate
	I am confident I can explain something about digital forensics to a family member or friend	+ 1.302	0.994	< 0.001	1.31	Large
	Digital forensics relates to what I experience in the real world	+ 0.746	0.822	< 0.001	0.91	Large
	I would like to learn more about careers in computer science like digital forensics and cybersecurity	+ 0.587	0.835	< 0.001	0.70	Moderate
Males (n = 75)	I am interested in computer science topics	+ 0.400	0.771	< 0.001	0.52	Moderate
	I am interested in digital forensics topics	+ 0.520	0.964	< 0.001	0.54	Moderate
	I am interested in using digital forensic applications to solve problems	+ 0.720	1.085	< 0.001	0.66	Moderate
	I am confident I can explain something about digital forensics to a family member or friend	+ 1.080	1.037	< 0.001	1.04	Large
	Digital forensics relates to what I experience in the real world	+ 0.800	0.986	< 0.001	0.81	Large
	I would like to learn more about careers in computer science like digital forensics and cybersecurity	+ 0.387	0.899	< 0.001	0.43	Small

For all statements, both males and females saw gains that were statistically significant and with mostly moderate to large effect sizes. One notable difference in the strength of the effect size based on gender was the question “I would like to learn more about careers in computer science like digital forensics and cybersecurity,” where the effect is size was moderate for females and small for males. In contrast, the statement “I am interested in computer science topics” showed the inverse, with a small effect size strength for females, and moderate for males. These findings suggest that both males and females are benefitting equally from the CSSL experience in terms of interest in and understanding of digital forensics as well as seeing the relevance of digital forensics to their everyday life. When asked about their interest in learning more about related careers, girls expressed a more significant rise in interest than boys after participating in the CSSL program.

The independent samples t-test comparing by gender of pre-post survey results related to research questions are presented in Table 4 below.

**Table 4:** Independent samples t-test comparison by gender of students’ understanding of and interest in digital forensic science and related careers based on retrospective pre-post survey questions

Timepoint	Statement	Gender	Mean (Scale: 1–4)	SD	*p*-value	Effect size (d)	Effect size strength
Retrospective pre (n = 63 females, 75 males)	I am interested in computer science topics	Female	2.60	0.908	0.025	0.39	Small
		Male	2.95	0.868			
	I am interested in digital forensics topics	Female	2.37	0.848	0.900	N/A	N/A
		Male	2.35	0.862			
	I am interested in using digital forensic applications to solve problems	Female	2.37	0.809	0.825	N/A	N/A
		Male	2.33	0.859			
	I am confident I can explain something about digital forensics to a family member or friend	Female	1.65	0.722	0.048	0.35	Small
		Male	1.95	0.971			
	Digital forensics relates to what I experience in the real world	Female	2.35	0.901	0.810	N/A	N/A
		Male	2.39	0.914			
	I would like to learn more about careers in computer science like digital forensics and cybersecurity	Female	2.33	0.861	0.104	N/A	N/A
		Male	2.57	0.857			
Post (n = 63 females, 75 males)	I am interested in computer science topics	Female	2.92	0.955	0.003	0.52	Moderate
		Male	3.35	0.688			
	I am interested in digital forensics topics	Female	2.87	0.871	0.965	N/A	N/A
		Male	2.87	0.827			
	I am interested in using digital forensic applications to solve problems	Female	2.94	0.821	0.416	N/A	N/A
		Male	3.05	0.853			
	I am confident I can explain something about digital forensics to a family member or friend	Female	2.95	0.906	0.614	N/A	N/A
		Male	3.03	0.822			
	Digital forensics relates to what I experience in the real world	Female	3.10	0.875	0.518	N/A	N/A
		Male	3.19	0.783			
	I would like to learn more about careers in computer science like digital forensics and cybersecurity	Female	2.92	0.829	0.790	N/A	N/A
		Male	2.96	0.892			

Comparing retrospective pre-test and post-test means by gender, only a few differences were statistically significant. Females were less likely than males to come into the program with an interest in computer science and feeling confident they could explain something about digital forensics to someone else. Post-test scores show that females were no longer significantly different from males in their confidence explaining digital forensics; however, they were still significantly less likely than males to report an interest in computer science.

The independent samples t-test comparing by gender of pre-post survey results related to satisfaction questions are presented in Table 5 below.

**Table 5:** Independent samples t-test comparison by gender of students’ satisfaction based on retrospective pre-post survey questions

Statement	Gender	N	Mean (Scale: 1–4)	SD	*p*-value	Effect size (d)	Effect size strength
I understood how to use the Cyber Sleuth Science Lab digital platform	Female	61	2.98	0.741	0.584	N/A	N/A
	Male	75	3.05	0.733			
A digital platform like the one used in Cyber Sleuth Science Lab is a fun way to learn	Female	60	2.98	0.792	0.666	N/A	N/A
	Male	75	3.04	0.725			
I would like to participate in more programs like Cyber Sleuth Science Lab in the future	Female	61	2.70	0.882	0.713	N/A	N/A
	Male	75	2.76	0.852			
I would recommend Cyber Sleuth Science Lab to a friend	Female	60	2.90	0.681	.268	N/A	N/A
	Male	75	3.04	0.761			

Comparing four statements about their overall feelings about their CSSL experience, there were no significant differences between females and males. They were both equally likely to report that they understood how to use the CSSL digital platform, thought it was a fun way to learn, would like to participate in similar programs in the future, and would recommend CSSL to a friend. Therefore, the strategies for gender inclusive STEM instruction implemented in CSSL are effective for engaging girls without being harmful to boys’ engagement.

### Outcomes

A significant outcome of this research project is that the virtual learning environment developed in this project was highly effective for teaching digital forensic knowledge, skills, and abilities that are directly applicable in the workplace. This high effectiveness and engagement was evident throughout the project, through formative evaluations and in-class observations, as well as backend metrics showing the agility and speed with which the students acquired knowledge and skills, and progressed through the investigative tasks, despite the complex and challenging nature of the program. In addition, all participants developed a heightened awareness of security and privacy risks associated.

In addition, survey results and in-class observations across pilots found that girls relied more, relative to boys, on in-classroom supports than on supports embedded in the virtual learning environment. Girls actively engaged in group work and unplugged (outside of the computer) instructional activities such as structured in-classroom discussions, mock trial, Socratic circle, class timeline construction, interactions with female practitioner role models, rehearsing presentations and recording (e.g., FlipGrid for teacher feedback, report peer discussion and revision, etc.). Students who were engaged in the program particularly enjoyed the mock trial exercise examining the role digital evidence can play in a legal setting and discussing ethics in technology.

### Increased interest, understanding, and motivation in STEM

Another significant outcome of this research project is that the CSSL is effective for motivating underrepresented youth, particularly girls, to pursue education/careers in digital forensic science and cybersecurity in particular, and computer science in general. Across all pilots, in the aggregate, the majority of girls and boys who participated in the CSSL program were motivated and engaged by their experiences, and developed an increased knowledge and interest in digital forensic science and cybersecurity. There was an overall increase in interest among both girls and boys in using digital forensic tools to solve problems, and all students gained increased awareness of careers related to digital forensic science and cybersecurity and the experiences of professionals in these careers. Students were motivated and empowered by playing the role of investigators and getting hands-on experience with real-world digital forensic tools to help someone recover deleted data or deal with cyberbullying. An important aspect of this research outcome is that gender inclusive instruction in co-ed environments helps girls without discouraging boys.

One of the major outcomes of this research program was that, after participating in the CSSL program, girls expressed a more significant increase in interest than male students in learning more about careers related to digital forensics and cyber security.

In open ended survey questions, a primary motivation stated by girls was the direct relevance of the lessons to their daily lives, including online fraud and data privacy. Girls were more likely to mention personal connections to cyber safety, how it affects them personally, and what they would do differently (e.g., “[I] need to be more careful with passwords—I'll change mine after this course.”), while boys talked more about a general awareness of cybersecurity (e.g., “I didn't know that deleted messages could be found again”). These results are compatible with prior research findings that girls learning STEM are more motivated by real-world problems that have direct relevance to their lives, and are interested in protecting and serving their community using science and technology (Ashcraft et al., 2012; Christensen & Knezek, 2015; Diekman et al., 2015; Modi et al., 2012). Learning STEM through digital forensic science fulfills these motivations by addressing real-world problems and social issues such as safety, privacy, and justice.

A number of girls were interested in ongoing participation with the CSSL project, and are actively seeking educational opportunities in this field. One former student returned as a facilitator in 2019 and other students requested letters of support and recommendations as they pursue STEM related degrees.

### Cyber street smart and digital citizenship

This project embedded lessons about dealing with online risks and data privacy concerns. These lessons helped students develop cyber street smarts, including being better prepared to protect privacy, to practice safe/responsible behavior online, and to avoid problems in cyberspace (e.g., protecting personal information, preventing cyberbullying, resisting phishing attacks), and skills and knowledge to help others with problems including data recovery and digital forensic investigations. All participants in the CSSL developed a heightened awareness of security and privacy risks associated.

## Conclusions and future needs

The CSSL program provides a rich multidisciplinary learning experience, combining STEM with social issues. The virtual learning environment developed in this project is highly effective for teaching digital forensic knowledge, skills, and abilities that are directly applicable in the workplace. The CSSL was designed and developed to bring into practice the principles and strategies of effective gender inclusive instruction in informal STEM education. These supports included increasing in-classroom activities (e.g., mock trial, Socratic circle), group work for peer learning support, relevance of investigative scenarios to their daily lives, young female role-models and in-classroom facilitators (near peers). After participating in the CSSL program, girls expressed a significantly greater increase in interest, relative to boys, in learning more about careers related to digital forensics and cybersecurity.

Students enjoy learning in the virtual learning environment through investigative missions, and also benefit from “outside of the computer” activities such as structured in-classroom discussions, mock trials, and in-person interactions with practitioner role models. Girls favored such in-classroom activities more, relative to boys, who tend to prefer seeking supports in the virtual learning environment. Teaching STEM through digital forensic science taps into girls’ motivations to address real-world problems that have direct relevance to their lives, and to protect and serve their community. The strategies for gender inclusive STEM instruction implemented in CSSL are effective for engaging girls without being harmful to boys’ engagement.

Although the CSSL can be adapted to fit within different contexts and schedules, there are limits to this flexibility. The CSSL program is most effective when there is sufficient time for students to complete discrete activities. Although certain activities can be learned and completed in one hour, others require longer blocks of time, particularly complex technical tasks and longer activities such as a mock trial. Integrating the CSSL into a school curriculum requires a carefully arranged combination of hour-long sessions, some longer in class periods for complex technical tasks and mock trials, as well as assigned homework (e.g., report writing). When educators and facilitators had more time, they took the opportunity to incorporate additional materials that were of particular interest to them and the students, including references to current or local events. This had the added benefit of getting the facilitators more involved and engaged with the program and students.

As a future need, educators and facilitators expressed the need for additional professional development. On average, educators received 2 days of professional development prior to the CSSL program. Although this was sufficient for educators to become familiar with the platform and missions, they consistently expressed the need for more time to become familiar with the subject matter concepts and associated social issues. The educator in the Seattle 2019 pilot worked more closely with CSSL team members prior to implementation in order to learn subject matter and to adapt the curriculum for her class. This prolonged personal collaboration enabled the educator to be more confident and creative in the classroom, and established a strong relationship the CSSL team to provide support. Educators in other pilots had to learn on the job and had limited time to obtain support from the CSSL team, which made it more difficult to following the instructor guide for each module. In the future, if feasible, more comprehensive professional development will be created to give educators and facilitators an opportunity to learn about Digital Forensic Science and Cybersecurity, and to experience the entire program and platform from the perspective of a student. Teachers need more PD and ongoing community/material supports, both in the subject matter and gender inclusive instruction strategies. Teachers require additional time to explore the concepts/contexts, prepare for classroom implementation, and raise awareness of gender inclusive instruction practices such as providing girls with personal positive feedback and promoting a growth mindset. Such PD would increase teacher familiarity with the subject matter (concepts, processes and tools) and social context/impact, and the pedagogical approach for inquiry-based gender inclusive instruction.

This project identified needs/gaps in current curriculum that spawned the idea for future work that combines justice-oriented and culturally-responsive pedagogy and critical justice and expectancy-value theory to develop guided personalized learning pathways. Through this theoretical lens these individualized pathways could capitalize on underrepresented youth’s personal resources (i.e., lived experiences, funds of knowledge, personal community support network) to boost disciplinary learning, STEM identity development and industry community. This could strengthen community connections supported by technology with the goal of increasing the presence, persistence and impact of these students in the future workforce.

## Data Availability

Aggregate data provided in work.

## Abbreviations

CSSL: Cyber Sleuth Science Lab
IDLE: Investigate and Decide Learning Environment
NGCP: National Girls Collaborative Project
STEM: Science Technology Engineering Math
WANIC: Washington Network for Innovative Careers

## Code availability

Source code availability not applicable. Screenshots of user interface provided in work.
